# Ergot in Parturition—Enlarged Spleen

**Published:** 1876-05

**Authors:** A. J. Shaffer

**Affiliations:** Gainesville, Ga.


Gainesville, Ga., March 22, 1876.

*Dr. W. F. Westmoreland:*

Dear Sir—As your name has long been familiar to the medical student as one of the editors of the Atlanta Medical and Surgical Journal, and as one of its readers from the first number, in 1855, up to the present time, I do most heartily congratulate you and your associates upon having established it upon the firm rock of progressive medicine. As one of the most interesting features of the Journal is the reports of the sayings and doings of the Atlanta Academy of Medicine, and as the institution appears to have ignored all selfishness in those debates, it is to be hoped that the concentrated intelligence of the Academy will in a short time decapitate the tottering therapeutic reputation of that worthless drug, secale cornutum.

In my early practice I employed it freely, and thought it the *magnes bones* in re-establishing parturient uterine pains; but I finally suspected the little imposter of sporting with my credulity, and at last discarded it; for the last twelve years I

have not used it at all. I have no doubt that all the reputation that ergot has acquired as a uterine excitant is owing to the undue haste of accoucheurs, forgetting that the muscular contractions of the womb partake, in a great degree, of the same nature of other muscles, when excited by a local cause, that alternates seasons of pain and a time of rest; and as parturition is strictly a work of nature, she needs no untimely interference. As in all her other grand works, nature does not perform her duties according to the wishes of impatient man (by storm), but in accordance with her grand purposes. I have no doubt that many of the maladies common to women are caused by the hasty interference of accoucheurs, when called to parturient women; and especially is this the case with our younger brethren, as well as with some of the older members of the profession.

The following extreme cases will in part elucidate the advantages of not hastily interfering with nature's prerogative:

Case I.—In 1867 I visited Mrs. B., aged 32; a large, athletic woman, in her fourth confinement. May the 22d, at 10 o'clock P.M., her pains were active and regular. At 11 o'clock the membranes were ruptured, and the aqua amnii passed off in large quantities. The 23d, at 1 o'clock A.M., the pains entirely ceased; the vertex presenting, the os well dilated and dilatable. I left her at 7 A.M., sitting by the fire, and requested her to send for me when pain again set in.

On the 25th, at 2 o'clock P.M., pains recurred, and at 4 o'clock, she was delivered of twins. The mother and children did well.

Case II.—On June 15th, 1867, I visited Mrs. C., in her fifth confinement. For two hours the pains were regular and active; the os well dilated and dilatable; the vertex presenting. At 10 o'clock P.M. the membranes broke, the aqua amnii passed off in free gushes and in large quantities, after which the pains ceased. On the 17th, at 6 A.M., she was sitting quite comfortable. I left her, with the request that she would send for me as soon as the pains recurred. On the 20th, at 10 o'clock A.M., active pains recurred, and in two hours she was delivered of a healthy female child. Both mother and child did well.

Now in these cases, if I had made myself officious, I would have given ergot, or employed instruments, or might have done some other uncalled-for thing, and perhaps to the injury of the mother or child, or both.

But this is not what I intended to write when I commenced this letter, as my purpose was simply to record the following rare case of enlarged liver and spleen; but, impressed as I am with the many complications and dangers, both to mother and child, that are clearly traceable to meddlesome or unnecessary interference by the accoucheur, that I feel it the duty of every one not to permit an opportunity to pass to condemn the practice of interfering with the natural process of labor, except in cases where it is clearly and unmistakably demanded.

In 1862 I examined a boy, aged ten years, very anæmic, and the skin of a pale, ashy hue; weight sixty-five pounds. On examination of the abdomen, a large tumor was found, extending from the left iliac fossa upward and inclining to the left side, with the superior part resting against the diaphragm. The case was diagnosed to be unusual enlargement of the spleen. The liver was found to be quite large. I advised the father, Mr. Duncan, to punish the boy no longer with medicine, as a cure was impossible.

At the time of his death, November, 1862, he weighed seventy-five pounds. I conducted the autopsy, assisted by my friend Dr. T. K. Mitchel. At the time of his death his gross weight was seventy-five pounds; his clothing was at least five pounds. The spleen weighed sixteen and one-fourth pounds, made up principally of blood-vessels and a soft, friable tissue. The liver weighed eight and one-fourth pounds, of apparently healthy tissue. The gall bladder was normal in size, filled with healthy bile. The heart was slightly hypertrophied; the lungs were slightly atrophied, by reason of the enlarged abdominal viscera pressing upon the diaphragm, which reduced the calibre of the thorax. The kidneys were sound. The intellectual manifestations of the youth were rather bright.

It will be seen that the weight of the spleen and liver was a few pounds more than one-third the gross weight of the whole body. Yours truly,

A. J. Shaffer.

